# A Novel Dual-Modality Dual-View Hybrid Deep Learning–Machine Learning Framework for the Prediction of Carotid Plaque Vulnerability via Late Fusion

**DOI:** 10.3390/diagnostics16050807

**Published:** 2026-03-09

**Authors:** Wenxuan Zhang, Chao Hou, Xinyi Wang, Hongyu Kang, Shuai Li, Yu Sun, Yongping Zheng, Wei Zhang, Sai-Kit Lam

**Affiliations:** 1 Department of Biomedical Engineering, The Hong Kong Polytechnic University, Hong Kong SAR, China; wenxuan-wx.zhang@polyu.edu.hk (W.Z.); haylee-xinyi.wang@connect.polyu.hk (X.W.); hong-yu.kang@connect.polyu.hk (H.K.); sh.li@polyu.edu.hk (S.L.); stefanie.sun@polyu.edu.hk (Y.S.); yongping.zheng@polyu.edu.hk (Y.Z.); 2 Department of Ultrasound, Beijing Tiantan Hospital, Capital Medical University, Beijing 100069, China; houcdoctor@163.com; 3 Research Institute for Smart Ageing, The Hong Kong Polytechnic University, Hong Kong SAR, China

**Keywords:** carotid plaque, image classification, multimodal ultrasound imaging, stroke risk prediction, hybrid deep learning, machine learning

## Abstract

**Background**: Ultrasound imaging is an ideal tool for regular carotid plaque screening to identify individuals at high risk of stroke for clinical intervention. However, no existing study leverages multi-modal multi-view ultrasound imaging for AI-enabled auto-classification of carotid plaque vulnerability. This study aims to develop and validate an effective AI model for carotid plaque vulnerability classification through the applications of dual-modal (B-Mode and contrast-enhanced mode) dual-view (longitudinal and cross-sectional) settings to maximize the utility and potential of ultrasound imaging. **Methods**: Hybrid deep-learning (DL) and machine-learning (ML) methods were employed to balance between model discriminability and interpretability. B-Mode ultrasound (BMUS) and contrast-enhanced ultrasound (CEUS) images from 241 patients were retrospectively analyzed using the proposed hybrid-DL-ML variants. **Results**: Our findings suggest the hybrid VGG-RF model developed from a dual-modal dual-view setting outperforms those developed from other settings for identifying vulnerable carotid plaques. The VGG-RF model emerged as the best-performing model, achieving an optimal performance with an AUC of 0.908, precision of 0.765, recall of 0.929, specificity of 0.886, and F1 score of 0.839. The inherent interpretability of the VGG-RF model divulged that long-axis views of BMUS and CEUS images were the major contributing features for discriminating vulnerable carotid plaques against their counterparts. **Conclusions**: The present study underscored the effectiveness of AI models developed from dual-modal dual-view settings of ultrasound images. Notably, the hybrid VGG-RF model was benchmarked as the best-performing model among other studied hybrid DL-ML variants. Further studies on a larger cohort in a prospective setting are warranted to validate the findings of the current study.

## 1. Introduction

Stroke is one of the most prevalent aging-associated diseases worldwide [1]. Its incidence is rising rapidly with the ever-expanding aging population worldwide [2,3]. Carotid plaque, an accumulation of fat deposits and other materials in the carotid arteries, is highly associated with stroke occurrence and recurrence [4,5,6]. It triggers ischemic incidents in two ways: by constricting the artery and diminishing blood circulation or by detaching and obstructing a small artery within the brain [7]. The more vulnerable a plaque is, the more likely it is to detach and trigger a stroke. In light of this, there is a growing clinical interest in the community to deploy carotid plaque vulnerability as a biomarker for selecting and identifying high-risk patients ahead of their clinical presentation [8,9].

In clinical carotid plaque assessments, ultrasound imaging presents the most prevalent first-line and frequent surveillance tool among other imaging techniques, such as Magnetic Resonance Imaging (MRI), Computed Tomography (CT), and Positron Emission Tomography/Computed Tomography (PET/CT) [10,11]. Although MRI has become the gold standard for carotid plaque imaging owing to its superior image resolution and detection sensitivity, the applicability of MRI has been largely restricted by its nature of low accessibility, low affordability, and its contraindication to patients with metallic implants or claustrophobia [12]. CT provides high-resolution imaging and identification of ulceration and calcification; notwithstanding, its nature of radiation hazards has posed a significant barrier for routine, regular, frequent, and population-wide screening for carotid plaque. Similarly, although PET/CT could be effective to detect active inflammation within the plaque, it shared similar deficiencies as CT imaging on top of its shortcomings in assessing plaque characteristics, such as ulceration, intraplaque hemorrhage, or lipid-rich necrotic core. By contrast, ultrasound imaging is a cost-effective approach for evaluating plaque morphology and characteristics, and it offers sufficient resolution for carotid plaque detection and assessments. Furthermore, ultrasound scanners are more portable, financially affordable, and free of ionizing radiation hazards [13,14]. These unique features of ultrasound imaging have been increasingly consolidating its role as an ideal imaging tool for routine, regular, repeated, and large-scale population-wide screening and identification of high-risk individuals for stroke.

Despite the abovementioned superiorities, plaque assessment via ultrasound imaging is still challenging due to artifacts, noises [15,16], and observer variability [17]. For this reason, AI-assisted methods are in great demand for ultrasound imaging-based carotid plaque assessment. Moreover, the potential of ultrasound imaging in the domain of AI-assisted carotid plaque assessment could be further strengthened by leveraging its multi-modality (e.g., BMUS, CEUS) and multi-view (e.g., longitudinal view, cross-sectional view). Over the past decades, multi-modality/multi-view analysis has shown promising results in the clinical field compared to single-modal/single-view imaging [18,19]. Additional modalities beyond conventional ultrasound imaging, i.e., BMUS, could provide extra information on the research topic concerned, enhancing the understanding of its underlying mechanism. For example, a recent study by Cheng et al. (2023) [20] employed a set of features extracted from Shear Wave Elastography (SWE) and Superb Microvascular Imaging (SMI) on carotid plaque. In their study, SWE provided tissue stiffness information, and SMI provided insights into neovascularization, leading to its efficacy in plaque condition assessment and prediction of short-term outcome after stroke. Although their findings are encouraging, SWE and SMI have not been widely adopted in routine clinical practice compared to BMUS and CEUS. Another study by [21] leveraged CEUS to detect neovascularization inside plaques. In addition, multi-view analysis facilitates the acquisition and interpretation of data from diverse vantage points, thereby enriching the comprehensiveness of the research investigation. For example, Ref. [22] employed front and side views of neck region photographs in their radiation dermatitis severity grading network, resulting in a comparable performance to human assessors with higher efficiency. In the case of carotid plaques, a longitudinal view provides characteristics of the length and morphology of the plaque, while a cross-sectional view offers insights into the depth and composition. As such, it is pertinent that these views may work in tandem to provide complementary information for carotid plaque vulnerability in the context of AI.

Notably, there is no existing study in the current body of literature leveraging a multi-modal multi-view of ultrasound images to maximize its utility and potential for AI-enabled auto-classification of carotid plaque vulnerability. This may be ascribed to the practical challenges in obtaining both modalities for each individual and obtaining accurate manual delineations of carotid plaques by experienced clinicians for all the studied ultrasound imaging modalities, which itself could be an overwhelmingly resource- and expertise-demanding procedure. On top of that, most of the existing models are either based on DL algorithms that are highly predictive but of low interpretability due to the intrinsic properties of DL [23,24] or based on ML algorithms that are interpretable but with limited discriminability due to their relatively inferior classification power when compared to DL [25,26]. Of note, there is a severe lack of studies combining both DL and ML for achieving a balance between model discriminability and interpretability in the context of carotid plaque vulnerability classification.

Confronted with this, the present study aimed to bridge this gap by benchmarking the best-performing hybrid DL-ML model that leverages dual-modal (BMUS and CEUS) and dual-view (longitudinal view and cross-sectional view) ultrasound images for discriminating vulnerable carotid plaques against their counterparts. Key contributions of this study include the following:

1. This study proposed a novel dual-modal dual-view DL-ML framework, which enables automatic classification of carotid plaque vulnerability on B-Mode and contrast-enhanced ultrasound images with longitudinal and cross-sectional views.
2. To overcome the data variety introduced by the dual-modal dual-view data setting, a late fusion hybrid DL-ML method was proposed to effectively fuse features extracted from different ultrasound image modalities and views.
3. This research benchmarked the hybrid VGG-RF model as the best-performing model among all model variants, with an AUC of 0.908, a precision of 0.785, a recall of 0.953, a specificity of 0.895, and an F1 score of 0.865. Its performance is comparable to peer models trained on a single-view, single-modality settings, i.e., BMUS longitudinal view on larger cohorts [24].
4. To our best knowledge, the first of its kind to use bounding box ROI in US-based plaque vulnerability prediction. This strategy offers an alternative to the delineation ROI definition adapted by prior studies [23,24,27]. It broadens accessibility across imaging modalities, mitigates potential information loss from indistinct boundaries, and enhances ease of use for clinicians.

## 2. Materials and Methods

### 2.1. Patient Data

**Figure 1:**
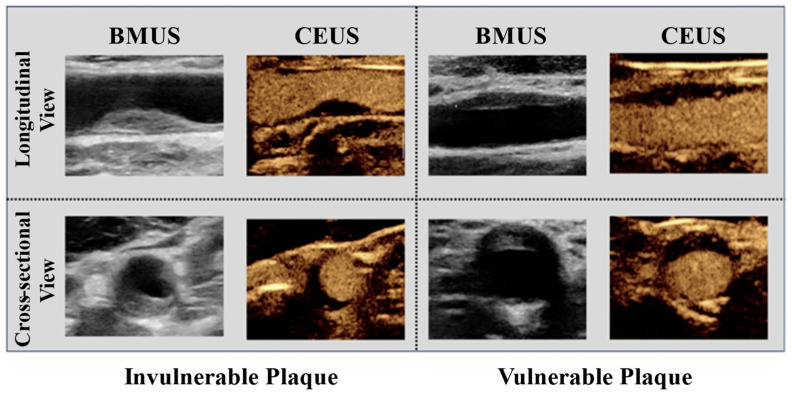
Sample patient data.

Patient data were retrospectively retrieved from Beijing Tiantan Hospital between 2014 and 2022. Ethical approval was obtained from the Human Subject Ethics Sub-committee of the Hong Kong Polytechnic University (HSEARS20240719004) and from the Ethics Board of Beijing Tiantan Hospital, Capital Medical University (No. KT2022-015-04). BMUS and CEUS images from 241 patients were included in this study after screening 1088 subjects in total. Both longitudinal and cross-sectional views of BMUS and CEUS were analyzed as shown in Figure 1. For each patient sample, a single representative image was selected per view to ensure consistency. The images were acquired from two different ultrasound scanners. The first scanner was a Canon Aplio 900 (Canon Medical Systems Corporation, Ōtawara, Tochigi, Japan) with a 10–15 MHz i18LX5 transducer. The second scanner was a Supersonic Imagine Aixplorer (SuperSonic Imagine, Aix-en-Provence, France) with a 6–9 MHz SL10-2 transducer. A bounding box region-of-interest (ROI) was generated to cover the carotid plaque on the images for downstream analysis. Ground truth for plaque vulnerability was provided by experienced physicians through consensus at Beijing Tiantan Hospital.

### 2.2. Design of the Hybrid DL-ML Model

In this study, a total of four types of input data were processed in the proposed dual-modal dual-view image fusion networks. They are BMUS longitudinal view (BMUS-L), BMUS cross-sectional view (BMUS-C), CEUS longitudinal view (CEUS-L), and CEUS cross-sectional view (CEUS-C), as shown in Figure 1.

Multimodal fusion methods can be broadly classified into early fusion and late fusion approaches, depending on when the fusion process is introduced. Early fusion refers to concatenating and jointly learning features within the network, which can capture cross-modal interactions but may be sensitive to modality imbalance and noise. Late fusion, in contrast, combines complementary outputs from separate networks at the decision level, generating a joint prediction. Beyond these two categories, recent reviews highlight intermediate and hybrid strategies, such as hierarchical fusion and attention-based fusion, which aim to balance feature-level integration with decision-level robustness [28]. In medical imaging and cancer research, early fusion has been applied to integrate omics and imaging features, while late fusion has been favored when modalities differ substantially in scale or quality. Guided fusion strategies have also been proposed, where secondary data (e.g., clinical records) indirectly influence feature selection of primary data (e.g., imaging or omics) without direct concatenation.

In this study, late fusion was adopted to integrate dual-modality (BMUS and CEUS) and dual-view (longitudinal and cross-sectional) information. This design leverages the complementary strengths of each modality and view while maintaining flexibility and interpretability at the classifier level. Several considerations further motivated the choice of late fusion in this study:

1. Similar hybrid DL settings have been tested to be effective on carotid plaque classification tasks [24].
2. Given the retrospective design of this study, data from different modalities and views were not aligned on a real-time level or in exactly the same field of view (FOV). This, along with our dual-modal dual-view data setting, which introduced greater data variety, makes late fusion more suitable than early fusion. Early fusion could potentially impact performance due to these misalignments [29].
3. Based on the final classifier chosen, late fusion methods can enhance interpretability by highlighting which features from each modal/view contribute to the model.

Visual Geometry Group (VGG) [30] was chosen as a core neural network for deep feature extraction from each of the dual-modal dual-view ultrasound images. Ultrasound images primarily contain low-level anatomical and physiological information, such as tissue structures and blood flow. These features are relatively subtle and would benefit from applying deeper feature extraction mechanisms to divulge imaging biomarkers. Therefore, VGG was chosen owing to its capability of extracting deep features from images. Sanagala et al.’s [23] work also showed that VGG was the most effective model for the transfer learning method on carotid plaque images among various models. Pretrained weights obtained from ImageNet [31] were loaded to speed up the training process.

**Figure 2:**
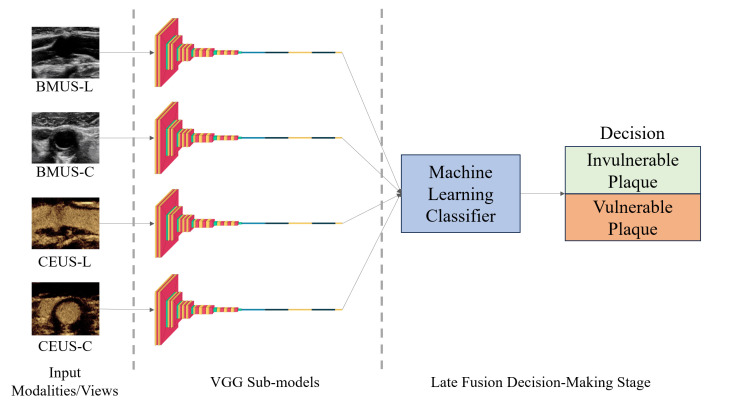
Model structure.

Subsequently, six ML algorithms were independently applied to the output features derived from the VGG models, serving as their inputs to fit each of these classifiers. These algorithms include Decision Tree (DT), K-Nearest Neighbors (KNN), Random Forest (RF), Support Vector Machines (SVMs), Logistic Regression (LR), and Light Gradient Boosting Machine (LGBM) [32]. A general schematic diagram for the proposed hybrid DL-ML models is illuminated in Figure 2.

### 2.3. Experimental Protocol

#### 2.3.1. Data Split

The dataset contains 241 samples (55 positive, 186 negative). Patient-level splitting was strictly enforced so that no patient appeared in more than one set within a fold. To evaluate model performance, a stratified 5-fold cross-validation was conducted on the whole dataset. In each fold, the dataset was partitioned into training and testing sets with a ratio of 80/20, yielding training sets of 192 samples (41 positive, 151 negative) and test sets of 49 samples (14 positive, 35 negative). Stratification preserved the ratio of positive and negative cases in every fold. For the VGG network, validation sets were further split from the training sets in each fold with a ratio of 80/20, resulting in validation sets of 39 samples (5 positive, 34 negative) and the remaining 153 samples (36 positive, 117 negative) used for training. This procedure was designed to prevent data leakage and provide a more stable estimate of model generalizability in the context of a relatively small and imbalanced dataset.

Although the dataset is imbalanced, with approximately 23% vulnerable plaques, this distribution closely reflects real-world prevalence. Resampling strategies (e.g., SMOTE) were not applied to the training set because the limited number of minority cases may yield synthetic samples with low diversity, risking overfitting and inflated performance [33,34,35]. Down-sampling may further discard the majority-class patients, which could be problematic for CNN training stability. Instead, several classifiers (RF, SVMs, LR, LGBM) incorporate adaptive class weighting, which partially mitigates imbalance effects without altering the dataset distribution.

#### 2.3.2. Model Development

Firstly, four distinct VGG models, each representing a specific modality and view, were trained independently. These models were trained on the BMUS-L, BMUS-C, CEUS-L, and CEUS-C data from the training set, respectively. The validation set was used to optimize the performance and guide the training process of each of these models. Subsequently, the logits of the last layer before prediction from the four VGG models were loaded. These logits were then sent into various ML models, including DT, KNN, RF, SVMs, LR, and LGBM, to fuse the complementary features between different modalities and views of input ultrasound images. For the VGG network, the Adam optimizer was employed with categorical cross-entropy loss. These settings are consistent with widely adopted defaults in deep learning frameworks and have been shown to provide stable convergence in medical imaging tasks, particularly when datasets are relatively small and imbalanced [36,37]. A fixed learning rate of 1 × 10^−4^ was used rather than a decay schedule, as this choice offered stable training dynamics and reproducibility in the context of a limited dataset. For the machine learning classifiers, hyperparameters were selected using grid search with cross-validation on the training set. This procedure ensured that parameter tuning was systematic and reproducible while reducing the risk of overfitting to a particular partition [38,39]. The best-performing parameter set identified through this process was then used for subsequent evaluation. This approach was adopted to fully leverage the potential of the ML classifiers and to enable a fair comparison with the deep learning models under consistent evaluation conditions.

To evaluate the effectiveness of the developed models, four experimental settings were adapted to train on different input combinations, namely single-modal single-view, single-modal dual-view, dual-modal single-view, and dual-modal dual-view. Their input combinations are shown in Table 1. The models developed in the single-modal single-view setting were regarded as baselines for model performance comparisons against models developed in other experimental settings. The models generated in the single-modal dual-view and dual-modal single-view settings were employed to provide an evaluation of model performance against the dual-modal dual-view models, which leverage complementary imaging features from all modalities and views of ultrasound images.

#### 2.3.3. Model Evaluation

A thorough evaluation of models was conducted on the testing set, which was developed based on different combinations of modalities and views: single-modal single-view, single-modal dual-view, dual-modal single-view, and dual-modal dual-view. The primary metrics for this evaluation were the AUC and F1 score. To quantify the variability of model performance, 95% confidence intervals (CIs) were calculated for AUC and F1 scores based on fold-level results from cross-validation. The DeLong test [40] was utilized for comparisons between ROC-AUC curves of different models. A *p*-value less than 0.05 indicates statistical significance of the performance differences between the ROC-AUC curves compared.

The best-performing hybrid DL-ML variant was then benchmarked using the AUC and F1 score on the testing set. This model was further evaluated in terms of precision, sensitivity, and specificity. Additionally, model interpretability was assessed by SHAPLEY values [41,42], which revealed the average contributions of each feature to the model output. Complementary qualitative insights into CNN feature extraction were obtained for each modality and view through Grad-CAM visualizations [43]. This comprehensive evaluation process ensured that both the average performance and the variability/stability of the model performance were assessed, providing a comprehensive understanding of the proposed models’ effectiveness.

## 3. Results

### 3.1. Comparative Analysis of Experimental Settings

**Figure 3:**
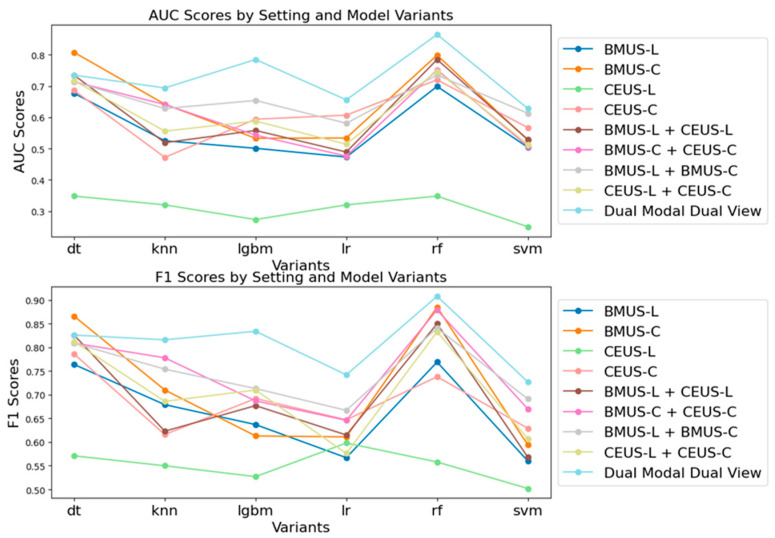
Comparison of AUC and F1 scores on different experimental settings and model variants.

Table 2 and Table 3 depict the ROC-AUC values and F1 scores, respectively, of the models developed in different experimental settings. Figure 3 shows the comparison of AUC and F1 scores on different experimental settings and model variants. Three key findings were generated:

Enhancement through modality and view fusion: The experimental results indicated that the fusion of either two modalities (dual-modal single-view) or two views (single-modal dual-view) generally leads to an improvement in model performance compared to the respective baselines (e.g., BMUS-L + CEUS-L compared to BMUS-L and CEUS-L). This suggests that the integration of additional modalities or views of ultrasound images can effectively enhance the predictive power of the deep learning models.

Superiority of dual-modal dual-view setting: The dual-modal dual-view setting, which incorporates all available modalities and views, consistently outperformed other settings across most models except VGG-DT and VGG-KNN, as reflected by the AUC and F1 scores. This finding substantiates our hypothesis that a comprehensive fusion of multi-modal multi-view images could potentially optimize the performance of AI models using carotid plaque ultrasound imaging.

Optimal performance of the VGG-RF model: Among the various hybrid DL-ML variants, the VGG-RF (VGG with Random Forest) model demonstrated superior performance across all settings (AUC: 0.908; F1 score: 0.865). This underscores the effectiveness of the VGG-RF model in leveraging multi-modal multi-view data for enhanced predictive accuracy.

**Figure 4:**
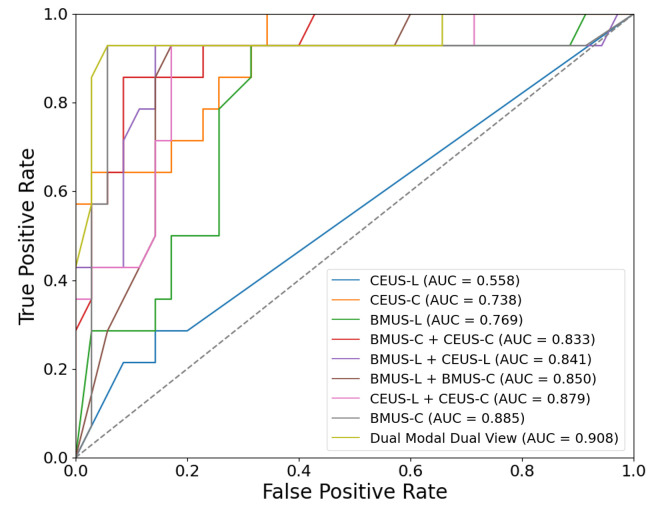
ROC-AUC curves of the hybrid VGG-RF model on different experimental settings.

DeLong tests were performed to evaluate the statistical significance of the difference between each ROC-AUC curve. As shown in Table 4, the majority of the comparisons yielded *p*-values less than 0.05, indicating statistically significant differences in performance between dual-modal dual-view and other settings. The ROC-AUC curves are shown in Figure 4. However, several comparisons (e.g., vs. BMUS-C and vs. BMUS-C + CEUS-C) did not reach statistical significance. These exceptions suggest that while the dual-modality dual-view approach generally provides improved performance, its superiority is not universal across all settings. Nonetheless, the overall performance metrics and the majority of significant comparisons support the practical value of dual-modal dual-view fusion in plaque vulnerability classification. The overall DeLong test results for all models and settings are attached in appendices, as shown in Table A1, Table A2, Table A3, Table A4, Table A5 and Table A6.

### 3.2. Benchmarking the Optimal Hybrid DL-ML Variant

The VGG-RF model consistently demonstrated superior performance in a dual-modal, dual-view setting. This is evident in the AUC values and F1 scores presented in Table 2 and Table 3, respectively. The VGG-RF model achieved an outstanding AUC value of 0.908 in a dual-modal dual-view setting (BMUS-L/C + CEUS-L/C), which yielded the highest AUC value across all models and settings. Meanwhile, it also achieved the highest F1 score of 0.865 in a dual-modal dual-view setting, underscoring the effectiveness of the VGG-RF model in leveraging multi-modal multi-view data for enhanced predictive accuracy.

### 3.3. In-Depth Evaluation and Interpretability of the Benchmarked Model

The performance metrics of the VGG-RF model across different experimental settings are presented in Table 5. The metrics include AUC, precision, recall, specificity, and F1 score. The dual-modal dual-view setting, specifically with the combination of BMUS-L/C + CEUS-L/C, demonstrates superior performance. This is evidenced by an AUC of 0.908, precision of 0.785, recall of 0.953, specificity of 0.895, and an F1 score of 0.865. These metrics indicate a high level of effectiveness in this particular setting.

**Figure 5:**
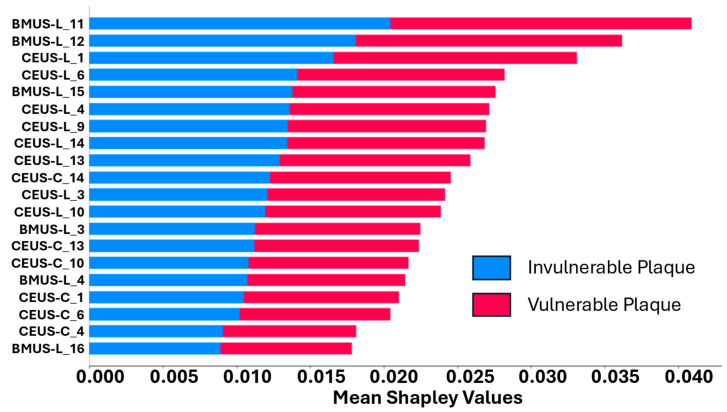
Average feature impact on model output magnitude of the hybrid VGG-RF model.

As for model interpretability, Figure 5 displays the SHAPLEY values for each of the selected imaging features in the benchmarked VGG-RF model. It shows that BMUS-L exhibits the most significant influence on the model’s classification decision, followed by CEUS-L. Notably, while CEUS-L alone demonstrates poor predictive performance with an AUC of 0.558, it significantly enhances the overall model performance when combined with other features. This highlights the effectiveness of modality fusion in improving predictive accuracy. The major contribution comes from the longitudinal view of ultrasound images (i.e., BMUS-L and CEUS-L), indicating that features from longitudinal views are crucial in determining the predictive outcomes of the VGG-RF model when compared to cross-sectional views, offering valuable insights into how the model makes its decisions in classifying plaque vulnerability. However, it should be noted that this interpretability analysis is partial: SHAP explains the contribution of imaging features within the ML classifiers but does not directly account for the CNN feature extraction process.

**Figure 6:**
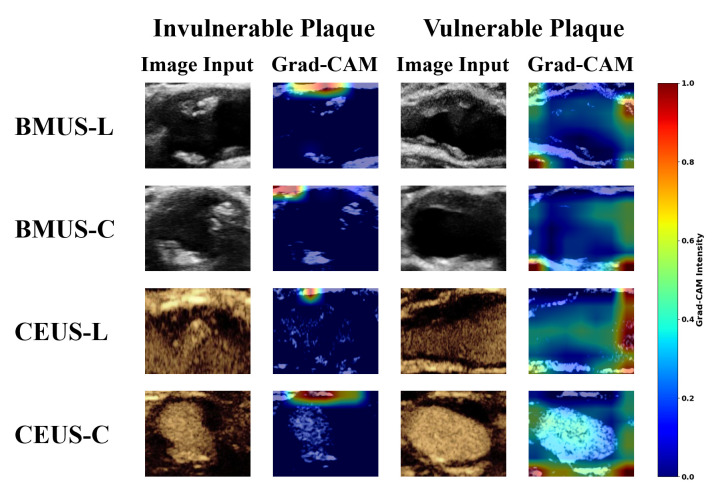
Representative cases of Grad-CAM visualizations of ultrasound images across modalities and views for invulnerable and vulnerable plaques.

As shown in Figure 6, complementary qualitative insights were obtained through Grad-CAM visualizations for each modality and view from the trained VGG models. For invulnerable plaques, the model’s attention was primarily concentrated along the upper boundary of the vessel, which may suggest reliance on surface morphology cues, consistent with guideline descriptions of stable plaque features [13]. In contrast, for vulnerable plaques, Grad-CAM highlighted more nuanced regions within the vessel lumen and surrounding structures, which may reflect the irregular boundaries and heterogeneous textures often reported in unstable plaques [44].

## 4. Discussion

This study pioneers the use of dual-modal dual-view ultrasound images for AI-enabled carotid plaque classification. It demonstrated the effectiveness of the proposed approach with the dual-modal, dual-view setting that outperformed other experimental settings compared in the present study. Notably, the VGG-RF model emerged as the best-performing network among all the hybrid DL-ML variants studied, achieving an optimal performance with an AUC of 0.908 in the testing set, along with precision of 0.785, recall of 0.953, specificity of 0.895, and an F1 score of 0.865, suggesting that the benchmarked model is a promising network for carotid plaque classification on the basis of dual-modal dual-view ultrasound images. The inherent interpretability of the RF was embedded in the VGG-RF model, divulging that a longitudinal view of BMUS and CEUS images was the major contributing feature for discriminating vulnerable carotid plaques against their counterparts.

### 4.1. Model Discrepancies

From the results in Table 2, the VGG-DT model achieved an AUC score of 0.866 in a single-modal, single-view setting with the input BMUS-C. This performance deviates from the general trend where models tend to perform better with the integration of additional modalities or views. DT, being relatively straightforward models, are well-suited for less intricate data. The incorporation of additional modalities or views may introduce a level of complexity that the decision tree model might struggle to handle effectively, potentially leading to diminished performance in these settings. Furthermore, DTs are prone to overfitting [45], which could explain their suboptimal performance in more complex settings.

Conversely, ensemble methods such as RF and LGBM have demonstrated superior performance across all models. These methods integrate multiple DTs, thereby capturing a wider range of interactions when fitting complex data. RF, in particular, introduces diversity into the model by using a subset of features for each tree and by bootstrapping the samples. This diversity could enhance the model’s robustness and generalization capability, making it more adept at handling the complexity introduced by additional modalities or views [46]. This observation suggests ensemble learning methods could be a promising direction for further exploration in multi-modal multi-view image analysis.

### 4.2. Benchmarking of the Hybrid DL-ML Variants

In this study, we elected to benchmark our proposed model, VGG-RF, against the work of [24] for two primary reasons. Firstly, Skandha et al.’s model represented the state-of-the-art (SOTA) in a single-modal single-view (SMSV) setting. Secondly, their model employed a hybrid DL structure that is analogous to the studied models in the current study, making it a relevant point of comparison. Performance benchmarking with state-of-the-art models is presented in Table 6.

The proposed VGG-RF model, trained on a dataset of 241 samples, achieved a mean AUC of 0.908 and a recall of 0.953. The CNN-DT model trained on the MixD dataset (2600 samples) reported a mean AUC of 0.983 and a recall of 0.983, while the LisD cohort (1500 samples) achieved an AUC of 0.825 and a recall of 0.880. These results demonstrate variability in performance across datasets of different sizes and levels of curation. Additionally, a trend was observed in the performance of the CNN-DT model: as the size of the training dataset increased, the model’s performance also improved. In contrast, the VGG-RF model was trained on a smaller, retrospective dataset, which may limit generalizability. Moreover, both LonD and LisD were dedicatedly designed for plaque tissue classification research [25,47] while this study employed a retrospective dataset. These distinctions of smaller sample size and differences in dataset curation underscore the need for cautious interpretation. Validation in larger, multi-center cohorts will be required to determine whether similar performance could be consistently reproduced and to assess the potential for further optimization.

### 4.3. Study Strengths

The primary novelty of this framework lies in its pioneering use of a dual-modal, dual-view approach, a strategy that has not been previously explored in the literature. This approach allows for a comprehensive interpretation of specific modalities and views of ultrasound images, thereby providing complementary clinical insights for physicians to identify at-risk patients ahead of their clinical presentation.

The second strength of this model is its hybrid deep learning-machine learning (DL-ML) structure, enabling satisfying model discriminability and model interpretability. The third notable advantage is the model’s stable performance despite being trained on retrospective data. This performance is comparable to models trained on larger, more specific datasets tailored for carotid plaque research.

Lastly, the use of a bounding box ROI in US-based plaque vulnerability prediction. Compared to the delineation ROI in existing studies [23,24,27], bounding box ROI has accessibility to more modalities. For example, in contrast-enhanced ultrasound, tissues would appear as a low-echo area. In such a case, a delineation of plaque tissues will contain very little info, while a bounding box ROI could provide context by including its surroundings. Also, a bounding box ROI may avoid potential information loss due to blurry boundaries of plaques caused by artifacts due to motion, air, or calcification tissues, and is easier to use for clinicians.

### 4.4. Limitations

The first limitation of this study is its retrospective nature in study design. This may lead to insufficient learning and uncontrollable factors such as types of modalities, patient clinical data, and acquisition settings. In addition, model evaluation was based on a single cross-validation scheme, which may limit the robustness of performance estimates. Future studies with larger, prospective datasets and more comprehensive validation strategies (e.g., repeated or nested cross-validation) are needed to strengthen the reliability of the findings and enhance the performance of the model.

Secondly, this study was conducted in a single center, which raises questions about the generalizability of the proposed model to other medical centers. Although scans in this study were acquired from multiple ultrasound vendors, no contrast normalization was applied, and the framework was evaluated directly on unprocessed CEUS images to reflect typical clinical practice. However, the absence of normalization may further limit generalizability, as inter-vendor variability was not addressed. A solution to this problem would be to conduct future studies across multiple centers, include more ultrasound machine vendors, and investigate whether normalization techniques could reduce variability and improve robustness [48,49].

Thirdly, this study was limited to certain imaging modalities, i.e., BMUS and CEUS, leaving the applicability to other imaging modalities untested. To address this, including modalities such as SWE and SMI in future studies is suggested [20,50,51]. Incorporating clinical information may also potentially enhance the model’s performance and interpretability, which is another potential area for improvement, as the current method utilizing SHAPLEY values only divulges feature contributions in ML classifiers and does not cover the DL part of the model.

In addition to these considerations regarding imaging modalities and the scope of SHAP-based feature attribution, another important limitation concerns the broader interpretability of the deep learning component. In this study, complementary qualitative insights were already explored through Grad-CAM visualizations, revealing distinct attention patterns between invulnerable and vulnerable plaques. While these visualizations highlight how CNNs focus on vessel boundaries or internal structures, such a visualization provides only partial interpretability. Building on this, future work should explore the application of Explainable AI (XAI) techniques [52,53,54,55,56]. These techniques aim to make the decision-making process of AI models more understandable to humans, which is crucial in the clinical field, as interpretability could be as important as accuracy.

Lastly, while bounding box ROI provides a practical alternative to delineation-based ROI, no direct experimental comparison with delineation-based ROI was performed in this study, and operator variability was not quantified. These aspects should be further investigated in future work.

## 5. Conclusions

In summary, this study underscores the effectiveness of modality and view fusion. Hybrid DL-ML models developed from dual-modal dual-view ultrasound images generally outperformed those developed from single-modal single-view, single-modal dual-view, and dual-modal single-view ultrasound images for identifying vulnerable carotid plaques. Among the studied variants, the hybrid VGG-RF framework achieved the strongest performance, with an AUC of 0.908, precision of 0.785, recall of 0.953, specificity of 0.895, and F1 score of 0.865. These results were obtained despite the relatively small retrospective dataset of 241 patients, underscoring the potential of the dual-modal dual-view integration strategy. Longitudinal views of BMUS and CEUS emerged as the most informative imaging features for discriminating vulnerable plaques in the proposed dual-modal dual-view framework.

Although the findings are encouraging, the retrospective design of the study and the small sample size may limit the strength of the results. Further prospective studies with larger cohorts are warranted to validate these findings. Multi-center studies to evaluate model generalizability to different medical centers and data acquisition settings, inclusion of additional modalities of ultrasound images and clinical patient information, and application of XAI techniques are also suggested in future studies.

## Figures and Tables

**Table 1:** Experimental settings.

Setting	Input Combination
Single-Modal Single-View	BMUS-L
	BMUS-C
	CEUS-L
	CEUS-C
Single-Modal Dual-View	BMUS-L + BMUS-C
	CEUS-L + CEUS-C
Dual-Modal Single-View	BMUS-L + CEUS-L
	BMUS-C + CEUS-C
Dual-Modal Dual-View	BMUS-L + BMUS-C + CEUS-L + CEUS-C

**Table 2:** ROC-AUC results with 95% CIs.

Experimental Setting	Input Combination	VGG-DT	VGG-KNN	VGG-LGBM	VGG-LR	VGG-RF	VGG-SVM
Single-Modal Single-View	BMUS-L	0.764 (0.685–0.843)	0.679 (0.556–0.802)	0.637 (0.576–0.698)	0.567 (0.479–0.655)	0.769 (0.646–0.892)	0.560 (0.481–0.639)
	BMUS-C	**0.866** (0.813–0.919)	0.710 (0.640–0.780)	0.613 (0.499–0.727)	0.611 (0.558–0.664)	0.885 (0.832–0.938)	0.594 (0.463–0.725)
	CEUS-L	0.571 (0.466–0.676)	0.550 (0.375–0.725)	0.527 (0.378–0.676)	0.598 (0.467–0.729)	0.558 (0.444–0.672)	0.502 (0.335–0.669)
	CEUS-C	0.786 (0.698–0.874)	0.616 (0.432–0.800)	0.692 (0.622–0.762)	0.647 (0.489–0.805)	0.738 (0.633–0.843)	0.629 (0.489–0.769)
Single-Modal Dual-View	BMUS-L + BMUS-C	0.826 (0.738–0.914)	0.623 (0.492–0.754)	0.677 (0.493–0.861)	0.615 (0.571–0.659)	0.850 (0.771–0.929)	0.568 (0.437–0.699)
	CEUS-L + CEUS-C	0.809 (0.774–0.844)	0.778 (0.655–0.901)	0.687 (0.617–0.757)	0.646 (0.506–0.786)	0.879 (0.774–0.984)	0.670 (0.521–0.819)
Dual-Modal Single-View	BMUS-L + CEUS-L	0.809 (0.756–0.862)	0.754 (0.649–0.859)	0.713 (0.573–0.853)	0.667 (0.536–0.798)	0.841 (0.771–0.911)	0.691 (0.577–0.805)
	BMUS-C + CEUS-C	0.812 (0.716–0.908)	0.686 (0.616–0.756)	0.710 (0.587–0.833)	0.576 (0.488–0.664)	0.833 (0.754–0.912)	0.607 (0.493–0.721)
Dual-Modal Dual-View	BMUS-L/C + CEUS-L/C	0.826 (0.730–0.922)	**0.816** (0.728–0.904)	**0.834** (0.755–0.913)	**0.742** (0.672–0.812)	**0.908** (0.882–0.934)	**0.727** (0.657–0.797)

Note: Bold font indicates the best-performing settings across experimental settings.

**Table 3:** F1 results with 95% CIs.

Experimental Setting	Input Combination	VGG-DT	VGG-KNN	VGG-LGBM	VGG-LR	VGG-RF	VGG-SVM
Single-Modal Single-View	BMUS-L	0.677 (0.624–0.730)	0.525 (0.429–0.621)	0.501 (0.405–0.597)	0.473 (0.385–0.561)	0.699 (0.655–0.743)	0.504 (0.408–0.600)
	BMUS-C	**0.808** (0.764–0.852)	0.642 (0.581–0.703)	0.533 (0.402–0.664)	0.534 (0.481–0.587)	0.799 (0.755–0.843)	0.527 (0.501–0.553)
	CEUS-L	0.348 (0.269–0.427)	0.320 (0.224–0.416)	0.273 (0.133–0.413)	0.320 (0.206–0.434)	0.348 (0.252–0.444)	0.250 (0.119–0.381)
	CEUS-C	0.686 (0.625–0.747)	0.472 (0.314–0.630)	0.594 (0.515–0.673)	0.607 (0.546–0.668)	0.720 (0.667–0.773)	0.567 (0.427–0.707)
Single-Modal Dual-View	BMUS-L + BMUS-C	0.735 (0.674–0.796)	0.519 (0.388–0.650)	0.558 (0.383–0.733)	0.489 (0.366–0.612)	0.785 (0.750–0.820)	0.529 (0.441–0.617)
	CEUS-L + CEUS-C	0.714 (0.679–0.749)	0.641 (0.588–0.694)	0.543 (0.438–0.648)	0.476 (0.397–0.555)	0.752 (0.691–0.813)	0.507 (0.428–0.586)
Dual-Modal Single-View	BMUS-L + CEUS-L	0.714 (0.670–0.758)	0.628 (0.523–0.733)	0.654 (0.566–0.742)	0.581 (0.520–0.642)	0.735 (0.665–0.805)	0.613 (0.490–0.736)
	BMUS-C + CEUS-C	0.717 (0.638–0.796)	0.556 (0.486–0.626)	0.588 (0.413–0.763)	0.514 (0.409–0.619)	0.748 (0.652–0.844)	0.513 (0.443–0.583)
Dual-Modal Dual-View	BMUS-L/C + CEUS-L/C	0.735 (0.665–0.805)	**0.694** (0.624–0.764)	**0.785** (0.724–0.846)	**0.656** (0.586–0.726)	**0.865** (0.830–0.900)	**0.628** (0.540–0.716)

Bold font indicates the best-performing settings across experimental settings.

**Table 4:** DeLong test results on the benchmarked VGG-RF model.

Setting 1	Setting 2	*p*-Value
BMUS-L/C + CEUS-L/C	BMUS-L	0.002
BMUS-L/C + CEUS-L/C	BMUS-C	0.124
BMUS-L/C + CEUS-L/C	CEUS-L	0.013
BMUS-L/C + CEUS-L/C	CEUS-C	0.035
BMUS-L/C + CEUS-L/C	BMUS-L + BMUS-C	0.031
BMUS-L/C + CEUS-L/C	CEUS-L + CEUS-C	0.026
BMUS-L/C + CEUS-L/C	BMUS-L + CEUS-L	0.079
BMUS-L/C + CEUS-L/C	BMUS-C + CEUS-C	0.223

**Table 5:** Performance metrics of the VGG-RF model across different experimental settings.

Experimental Setting	Input Combination	AUC	Precision	Recall	Specificity	F1 Score
Single-Modal Single-View	BMUS-L	0.769	0.526	0.976	0.648	0.699
	BMUS-C	0.885	0.674	0.929	0.819	0.799
	CEUS-L	0.558	0.421	0.262	0.857	0.348
	CEUS-C	0.738	0.500	**1.000**	0.600	0.72
Single-Modal Dual-View	BMUS-L + BMUS-C	0.85	0.659	0.953	0.800	0.785
	CEUS-L + CEUS-C	0.879	0.600	0.929	0.752	0.752
Dual-Modal Single-View	BMUS-L + CEUS-L	0.841	0.589	0.953	0.733	0.735
	BMUS-C + CEUS-C	0.833	0.589	0.953	0.733	0.748
Dual-Modal Dual-View	BMUS-L/C + CEUS-L/C	**0.908**	**0.785**	0.953	**0.895**	**0.865**

Bold font indicates the best-performing settings across experimental settings.

**Table 6:** Performance benchmarking of the proposed VGG-RF model with single-modality state-of-the-art models.

Model	Dataset	Data Size	AUC	Recall
VGG-RF (Proposed)	Retrospective Data	241	0.908	0.953
CNN-DT [24]	LonD	2000	0.945	0.961
	LisD	1500	0.825	0.88
	MixD	2600	0.983	0.983

LonD refers to the London dataset, and LisD refers to the Lisbon dataset. They are data used in [24]’s work. MixD is a combination of these 2 datasets. Metrics are compared in a balanced testing cohort.

## Data Availability

The dataset analyzed during the current study is available from the corresponding author on reasonable request, subject to patient privacy considerations. Trained VGG weights and machine learning pipelines used in this study are also available upon request to support reproducibility.

## Abbreviations

The following abbreviations are used in this manuscript:

AI	Artificial Intelligence
AUC	Area Under the Curve
BMUS	B-Mode Ultrasound
BMUS-L	B-Mode Ultrasound Longitudinal View
BMUS-C	B-Mode Ultrasound Cross-sectional View
CEUS	Contrast Enhanced Ultrasound
CEUS-L	Contrast Enhanced Ultrasound Longitudinal View
CEUS-C	Contrast Enhanced Ultrasound Cross-sectional View
CNN	Convolutional Neural Network
CT	Computed Tomography
DL	Deep Learning
DT	Decision Tree
FOV	Field of View
IPH	Intraplaque Hemorrhage
KNN	K-Nearest Neighbors
LGBM	Light Gradient Boosting Machine
LR	Logistic Regression
LRNC	Lipid-Rich Necrotic Core
ML	Machine Learning
MRI	Magnetic Resonance Imaging
PET/CT	Positron Emission Tomography/Computed Tomography
RF	Random Forest
ROC	Receiver Operating Characteristic
ROI	Region of Interest
SD	Standard Deviation
SWE	Shear Wave Elastography
SMI	Superb Microvascular Imaging
SVMs	Support Vector Machines
VGG	Visual Geometry Group (a type of convolutional neural network)

## Appendix A

**Table A1:** DeLong test results on the VGG-LGBM model.

Setting 1	Setting 2	*p*-Value
BMUS-L/C + CEUS-L/C	BMUS-L	0.047
BMUS-L/C + CEUS-L/C	BMUS-C	0.004
BMUS-L/C + CEUS-L/C	CEUS-L	0.001
BMUS-L/C + CEUS-L/C	CEUS-C	0.169
BMUS-L/C + CEUS-L/C	BMUS-L + BMUS-C	0.09
BMUS-L/C + CEUS-L/C	CEUS-L + CEUS-C	0.183
BMUS-L/C + CEUS-L/C	BMUS-L + CEUS-L	0.055
BMUS-L/C + CEUS-L/C	BMUS-C + CEUS-C	0.032

**Table A2:** DeLong test results on the VGG-RF model.

Setting 1	Setting 2	*p*-Value
BMUS-L/C + CEUS-L/C	BMUS-L	0.002
BMUS-L/C + CEUS-L/C	BMUS-C	0.124
BMUS-L/C + CEUS-L/C	CEUS-L	0.013
BMUS-L/C + CEUS-L/C	CEUS-C	0.035
BMUS-L/C + CEUS-L/C	BMUS-L + BMUS-C	0.031
BMUS-L/C + CEUS-L/C	CEUS-L + CEUS-C	0.026
BMUS-L/C + CEUS-L/C	BMUS-L + CEUS-L	0.079
BMUS-L/C + CEUS-L/C	BMUS-C + CEUS-C	0.223

**Table A3:** DeLong test results on the VGG-LR model.

Setting 1	Setting 2	*p*-Value
BMUS-L/C + CEUS-L/C	BMUS-L	0.032
BMUS-L/C + CEUS-L/C	BMUS-C	0.185
BMUS-L/C + CEUS-L/C	CEUS-L	0.184
BMUS-L/C + CEUS-L/C	CEUS-C	0.204
BMUS-L/C + CEUS-L/C	BMUS-L + BMUS-C	0.267
BMUS-L/C + CEUS-L/C	CEUS-L + CEUS-C	0.241
BMUS-L/C + CEUS-L/C	BMUS-L + CEUS-L	0.080
BMUS-L/C + CEUS-L/C	BMUS-C + CEUS-C	0.016

**Table A4:** DeLong test results on the VGG-DT model.

Setting 1	Setting 2	*p*-Value
BMUS-L/C + CEUS-L/C	BMUS-L	0.192
BMUS-L/C + CEUS-L/C	BMUS-C	0.367
BMUS-L/C + CEUS-L/C	CEUS-L	0.003
BMUS-L/C + CEUS-L/C	CEUS-C	0.602
BMUS-L/C + CEUS-L/C	BMUS-L + BMUS-C	0.709
BMUS-L/C + CEUS-L/C	CEUS-L + CEUS-C	0.811
BMUS-L/C + CEUS-L/C	BMUS-L + CEUS-L	1.000
BMUS-L/C + CEUS-L/C	BMUS-C + CEUS-C	0.766

**Table A5:** DeLong test results on the VGG-KNN model.

Setting 1	Setting 2	*p*-Value
BMUS-L/C + CEUS-L/C	BMUS-L	0.114
BMUS-L/C + CEUS-L/C	BMUS-C	0.295
BMUS-L/C + CEUS-L/C	CEUS-L	0.202
BMUS-L/C + CEUS-L/C	CEUS-C	0.046
BMUS-L/C + CEUS-L/C	BMUS-L + BMUS-C	0.044
BMUS-L/C + CEUS-L/C	CEUS-L + CEUS-C	0.616
BMUS-L/C + CEUS-L/C	BMUS-L + CEUS-L	0.757
BMUS-L/C + CEUS-L/C	BMUS-C + CEUS-C	0.025

**Table A6:** DeLong test results on the VGG-SVM model.

Setting 1	Setting 2	*p*-Value
BMUS-L/C + CEUS-L/C	BMUS-L	0.007
BMUS-L/C + CEUS-L/C	BMUS-C	0.061
BMUS-L/C + CEUS-L/C	CEUS-L	0.015
BMUS-L/C + CEUS-L/C	CEUS-C	0.099
BMUS-L/C + CEUS-L/C	BMUS-L + BMUS-C	0.052
BMUS-L/C + CEUS-L/C	CEUS-L + CEUS-C	0.622
BMUS-L/C + CEUS-L/C	BMUS-L + CEUS-L	0.604
BMUS-L/C + CEUS-L/C	BMUS-C + CEUS-C	0.051
